# A National Cross-Sectional Survey of Bullying in Syrian Graduate Medical Education

**DOI:** 10.3389/fpubh.2022.916385

**Published:** 2022-07-07

**Authors:** Sarya Swed, Sheikh Shoib, Mohammad Bader Almoshantaf, Haidara Bohsas, Ahmad Salah Eldin Mohamed Hassan, Karam R. Motawea, Noheir Ashraf Ibrahem Fathy Hassan, Eman Mohammed Sharif Ahmad, Lana Sheet, Lina Taha Khairy, Agyad Bakkour, Ali Hadi Hussein Muwaili, Dhuha Hadi Hussein Muwaili, Fatima Abubaker Abdalla Abdelmajid, Shoaib Ahmad, Mohammad Mehedi Hasan, Nashaat Kamal Hamdy Elkalagi

**Affiliations:** ^1^Faculty of Medicine, Aleppo University, Aleppo, Syria; ^2^Department of Psychiatry, Jawahar Lal Nehru Memorial Hospital, Srinagar, India; ^3^Department of Neurosurgery, Ibn Al-Nafees Hospital, Damascus, Syria; ^4^Resident of Plastic Surgery, Aswan University Hospital, Aswan, Egypt; ^5^Faculty of Medicine, Alexandria University, Alexandria, Egypt; ^6^Faculty of Medicine, Aswan University, Aswan, Egypt; ^7^Department of Obstetrics and Gynaecology, Nile Valley University, Atbra, Sudan; ^8^Faculty of Medicine, The National Ribat University, Al-Ribat, Sudan; ^9^Faculty of Medicine, Albaath University, Homs, Syria; ^10^Faculty of Medicine, Ivano-Frankivsk National Medical University, Ivano-Frankivsk Oblast, Ukraine; ^11^Faculty of Medicine, University of Medical Sciences and Technology, Al Khurtum, Sudan; ^12^District Head Quarters Teaching Hospital, Faisalabad, Pakistan; ^13^Faculty of Medicine, Punjab Medical College, Faisalabad, Pakistan; ^14^Department of Biochemistry and Molecular Biology, Faculty of Life Science, Mawlana Bhashani Science and Technology University, Tangail, Bangladesh; ^15^Internal Medicine and Tropical Medicine at Faculty of Medicine Al-Arish University, Al-Arish, Egypt

**Keywords:** bullying, medical graduates, victimization, cross-sectional, Syria

## Abstract

*Bullying* is defined as unpleasant behavior that causes someone to feel disturbed or embarrassed, affecting their self-esteem. Based on this premise, we set out to investigate bullying among Syrian graduate medical education residents and fellows, estimate its prevalence among specific subgroups, and give recommendations to help validate the findings and enhance the graduate medical education training experience. A sample of 278 residents and fellows in Syrian graduate medical school were recruited for the study in a national cross-sectional survey, with 276 participants completing a Bullying survey in 2021 and two people refusing to participate. Participants in the survey were asked to provide basic demographic and programming information and three general Bullying and 20 specific bullying behavior items. Differences across groups were compared for demographic and programmatic stratifications. About 51% of participants had experienced one or more bullying behaviors, 69% said they had been bullied, and 87% said they had witnessed Bullying. Residents and supervisor-attendings were the most common sources of perceived Bullying (~67 and 62%, respectively), followed by patients (58%), nurses (46%), and pharmacists (46%) (33%). More specific bullying behaviors have been recorded by female Arabic Syrians who are shorter than 5'8, have a body mass index (BMI) of 25, and are 30 years old or younger who were -compared to males- more likely to report attempts to minimize and devalue work (55 vs. 34%, *P* ≤ 0.01) and criticism and work monitoring (56 vs. 33%, *P* ≤ 0.01). In addition, general medical graduates and PGY-2-PGY-6 respondents reported more specific bullying behaviors than private medical graduates and post-graduate participants in the first year (PGY 1), respectively. For example, a significant difference is noticed when reporting unreasonable pressure to perform work (83 vs. 6%, *P* ≤ 0.01). Except for physical violence, which does not differ statistically between groups, most bullying behaviors were reported by participants with statistically significant differences between study groups—many residents and fellows in Syria's graduate medical school system report being bullied. Anti-bullying rules and a multidisciplinary strategy including all players in the medical system are essential to eradicating these pervasive practices in healthcare.

## Background

Bullying is described as unwelcome, aggressive behavior resulting from an actual or perceived social power imbalance (1). It can be repeated, or at the very least, has the potential to be repeated in the future (1). These behaviors are designed to make the victim feel uneasy. Bullying can take many different forms, including physical and verbal (2). Bullying has previously been proved to be pervasive in the medical school environment (2). It had been over three decades since the possibility of medical school abuse had been raised. Bullying is much more challenging when it comes to residents and fellows in the medical sector, which a resident is a practitioner who has finished medical school and is continuing their training in their desired field. At the same time, a fellow is a board-certified doctor who has completed medical residency and is continuing their education in their specialty. Doctors who have been bullied are believed to be less satisfied with their careers, take more sick days, and work fewer hours in the following 12 months due to their mistreatment (3).

Anti-bullying regulations and a multidisciplinary approach have been offered as measures to potentially prevent such behaviors in the healthcare business due to trainees' experiences with Bullying in graduate medical school programs in the United States (4). Daily, doctors-in-training face high academic demands, patient safety issues, and job insecurity. However, there is a high prevalence of abuse and neglect in schooling (3). According to a countrywide poll conducted by Daugherty et al. in the United States, over 93% of inhabitants had encountered at least one episode of perceived abuse. Embarrassment, sexual harassment, and discrimination have all been reported by some (4).

In Syria, the years of fighting have destroyed or damaged almost three-quarters of the country's hospital services. Further, there is a severe shortage of health care professionals. Thus, many Syrian doctors have fled to neighboring countries as refugees due to the lousy support. Therefore, it is essential to evaluate psychological support for the minority of doctors who did not immigrate.

There is reason to suspect that bullying in Syrian medical schools is still going unnoticed. In the Syrian community, let alone academia, “bullying” is not adequately defined. This study is the first national cross-sectional involving bullying in the Syrian graduate medical education system. This study aims to estimate the prevalence of bullying in the medical sector. This study will enable us to offer more efficient solutions and operate more competently to improve the educational and training experience.

## Methods

### Study Design, Setting, and Participants

The study was a cross-sectional survey conducted from 14 March to 20 April 2021, including residents and fellows in Syrian graduate medical education residing in the country; Data was collected from an online questionnaire published on several social media websites, including Facebook and WhatsApp, Twitter, and Telegram in Syria. The questionnaire was constructed from a previous American study (5) and then updated and translated to match the Syrian context. We used convenience and snowball as strategies to collect necessary data from the respondents. All medical residents and fellows were eligible to participate in the study, and the participation was voluntary with saving the personal information in a secure database.

Calcuator.com was used to calculate the sample size (http://calcuator.com/). According to the Syrian Ministry of Health's latest report (https://www.moh.gov.sy/), around 28,214 medical residents and fellows. We calculated the sample size using a statistical power analysis with a population percentage of 50%, a margin of error of 0.06, and a confidence level of 95%. The recommended sample size was 264. A sample of 278 residents and fellows in Syrian graduate medical education were invited to participate in this survey on the Google form website; two participants have refused. The final number of participants was 276.

### Measure

The questionnaire consists of two main parts; the first part was a range of questions about demographic data like age, gender, race, background in medicine, and position in the medical profession. The second section contains questions about workplace harassment and Bullying. *Workplace bullying* is a persistent, offensive behavior that causes the receiver to feel upset, threatened, embarrassed, or vulnerable, undermining their self-confidence. The second part is then subdivided into two subgroups; the first subgroup contains various questions about witnessing or experiencing, or being subjected to Bullying. For example: “In the past 12 months have you witnessed work colleagues being subjected to workplace bullying from peers, attendings, nurses, patients, or ancillary staff?” with answers ranging from “No” to “Frequently” it also contains a question to determine the source of Bullying either intern, supervisor, nurse, ancillary staff or patient, another question to determine the degree of health affection by bullying with answers ranging from “1” to “5.” The second subgroup includes questions participants should answer if they do not consider that they have been bullied, like “In the past 12 months, have you experienced from peers, attendings, consultants, nurses, patients or ancillary staff persistent attempts to belittle and undermine your work?” with answers ranging from “No” to “Frequently.”

### Statistical Analysis

The authors manually entered data from the formal written surveys into the original Google Form Online Questionnaire used to collect online data. Then the data was exported directly from the Google Form into an Excel spreadsheet. The raw data was then encoded in the Excel sheet to be used with the statistics program.

One way-ANOVA analysis was performed using the statistical package for social science version 25.0 (SPSS Inc., Chicago, IL, United States). Frequencies and percentages were used to describe categorical variables. The differences between baseline characteristic factors and bullying variables were investigated using one way-ANOVA test. *Statistical significance* was defined as a *P*-value <0.05.

### Ethical Considerations

The ethics committee approved the protocol of Aleppo University; additional ethical approval was taken from Ibn Al-Nafees Hospital. The aim of the present study was explained in the questionnaires, and informed consent was obtained from all the respondents through a Yes or No question inside the questionnaire asking participants whether they agreed to answer this questionnaire or not.

## Results

### Response Rate

A total of 275 replies were received, with 100% of participants completing the questionnaire, resulting in a 99% response rate.

### Demographics

Table 1 indicates the participants' age, gender, medical background, position in the medical profession, residency status, height, and BMI (BMI). Table 2 breaks down the data by specialization or sub-specialty.

**Table 1:** Baseline characteristics of the study sample (*N* = 276).

**Variables category**	**Number**	**Frequency**
Age		
30 and below	266	96.4%
30 and above	8	2.9%
Prefer not to say	2	0.7%
Gender		
Male	105	38%
Female	161	58.3%
Prefer not to say	10	3.6%
Background in medicine		
Graduate of private sector	22	8%
Graduate of general sector	246	89.1%
Prefer not to say	8	2.9%
Your position in the medical profession		
PGY*-1	84	30.4%
PGY-2- PGY-6	130	47.1%
Prefer Not to Say	62	22.5%
Residency status		
Arabic Syrian	242	87.7%
Arabic Non-Syrian	29	10.5%
Prefer not to say	5	1.8%
Your Height		
Under 5′ 8″	193	69.9%
Above 5′8″	79	28.6%
Prefer not to say	4	1.4%
Your Body Mass Index (BMI)		
Under 24.9	198	71.7%
Above 25.0	64	23.2%
Prefer not to say	14	5.1%

GY*-, Post Graduate Year.

**Table 2:** Summary of specialties in study sample (*N*= 276).

**Specialty**	**Number**	**Frequency**
Anaesthesiology	2	0.7%
Cardiology	9	3.3%
Emergency medicine	5	1.8%
Endocrinology	12	4.3%
ENT	25	9.1%
Family medicine	4	1.4%
General surgery	43	15.6%
Internal medicine	42	15.2%
Neurology	10	3.6%
Neurosurgery	9	3.3%
Ophthalmology	10	3.6%
Orthopedics	18	6.%
Pediatrics	11	4%
Psychiatry	1	0.4%
Pulmonology	7	2.5%
Radiology	4	1.4%
Urology	8	2.9%
Gynecology and obstetrics	56	20.3%

Ninety-six percent of those who responded were under the age of thirty. Males and females were unequally represented in the sample, with females accounting for 58.3% and males for 38%. Only 10.5% were Arabic non-Syrians, with Arabic Syrians accounting for the majority (87.7%). Most participants (89.1%) are general medical graduates, while just 8% are private medical graduates, with PGY-2–PGY-6 accounting for around 47% of completed questionnaires, while PGY-1 accounts for about 30%. The participants were unevenly distributed in terms of height, with around 70% under 5'8 and only 28.6% over 5'8. With a BMI of <24.9%, nearly 70% of the sample self-identified as being in a healthy weight range. Gynecology and obstetrics had the most remarkable response rate, followed by general surgery and internal medicine, with 18 specialties and subspecialties represented.

### Bullying Experiences in General and Differences Among Groups

**Figure 1:**
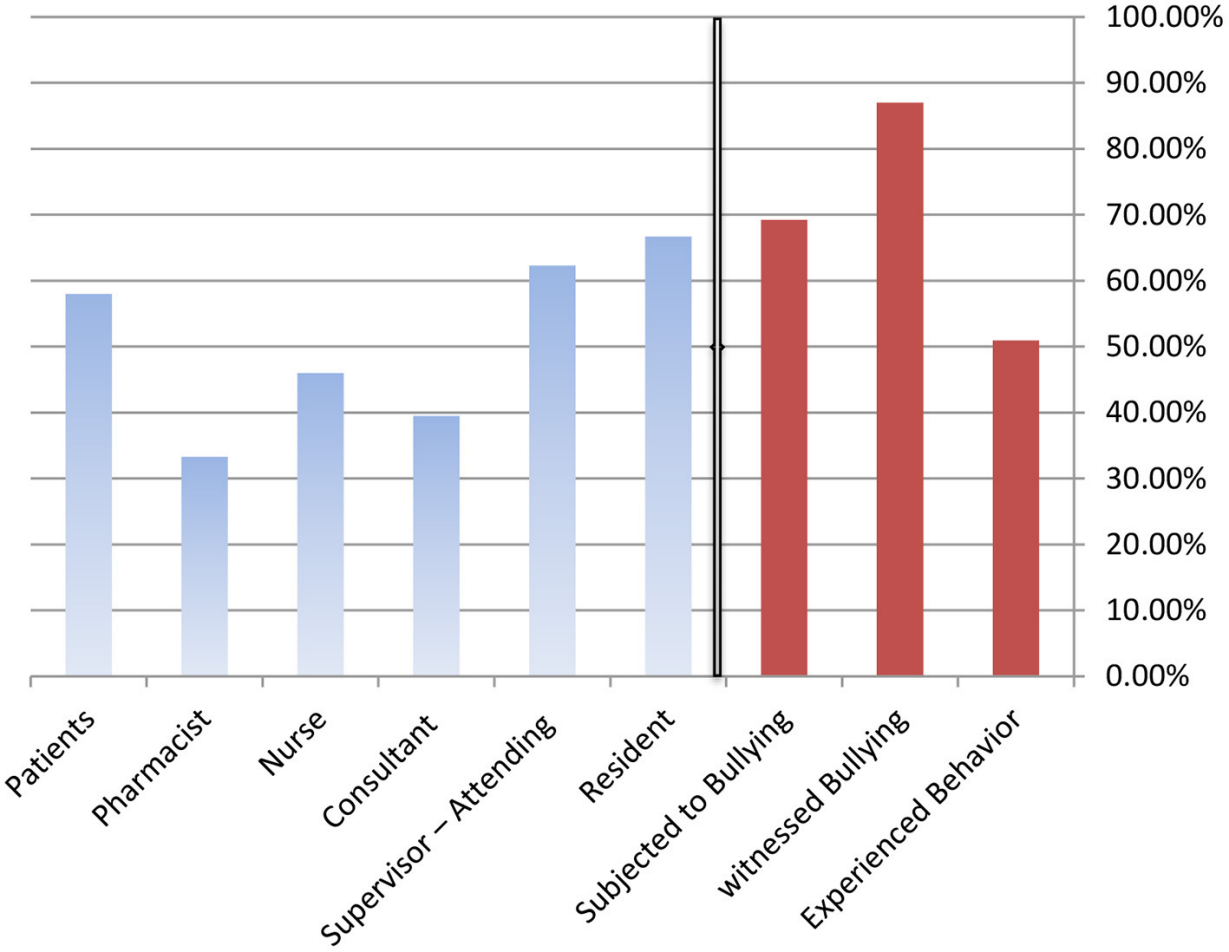
Percentage of participants who experienced bullying behaviors, witnessed bullying of colleagues, or were subjected to bullying themselves, and the source of that bullying.

Figure 1 depicts the percentage of those bullied, have observed or experienced bullying, and have been subjected to bullying themselves. “Experienced conduct” refers to when a participant indicates he or she has witnessed or been subjected to one or more of the specific bullying acts; however, “witnessed” and “subjected” were separate topics in the survey. Overall, around 51% of participants had experienced one or more of the bullying acts, 69% said they had been bullied, and 87% said they had observed bullying. The most common sources of reported bullying were residents and supervisor-attendings (~67% and 62%, respectively), followed by patients (58%), nurses (46%), and pharmacists (46%) (33%).

Bullying was more common among females (54%) than men (30%, *p* 0.01) and those under 30 years old (95%) than those over 30 years old (2%, *p* 0.01), with more females (54%) than males (30%, *p* 0.01). In addition, PGY-2-PGY-6 individuals were more likely to be bullied than PGY-1 participants (88 vs. 79%, *p* 0.01). Ethnicity, height, BMI categories, and general vs. private medical sector graduates had no statistically significant differences in the chance of being bullied.

### Specific Bullying Behaviors and Differences Among Groups

Participants were asked to indicate whether they had experienced 20 distinct bullying behaviors in addition to the overall bullying experience (Table 3).

**Table 3:** Percentages of bullying behaviors experienced by personal characteristics.

	**Total sample (*N* = 276)**.	**Gender**		**Ethnicity**		**Age**		**Height**		**BMI**	
		**Male (*n* = 105) 95%CI (lower-upper)**	**Female (*n* = 161) 95%CI (lower-upper)**	**Arabic Syrian (*n* = 242) 95%CI (lower-upper)**	**Arabic Non-Syrian (*n* = 29) 95%CI (lower-upper)**	**<30 (*n* = 266) 95%CI (lower-upper)**	**≥30 (*n* = 8) 95%CI (lower-upper)**	**<5′ 8″ (*n* = 193) 95%CI (lower-upper)**	**≥5′ 8″ (*n*= 79) 95%CI (lower-upper)**	**<25 (*n* = 198) 95%CI (lower-upper)**	**≥25 (*n* = 64) 95%CI (lower-upper)**
Subjected to bullying	88	30^**^ (0.72–0.88)	54 (0.88–0.96)	77 (0.84–0.92)	9 (0.73–1.00)	95^**^ (0.84–0.92)	2 (0.19–1.06)	64 (0.87–0.95)	26 (0.69–0.88)	77 (0.83–0.92)	9 (0.79–0.96)
attempts to belittle and undermine work	92	34^**^ (0.82–0.94)	55 (0.90–0.98)	81 (0.89–0.95)	10 (0.83–1.02)	89^**^ (0.89–0.95)	3 (0.57–1.17)	65 (0.89–0.96)	26 (0.83–0.96)	66 (0.88–0.96)	21 (0.85–0.98)
Persistent and unjustified criticism and monitoring of work	92	33^**^ (0.80–0.93)	56 (0.92–0.98)	81 (0.88–0.95)	10 (0.89–1.03)	90^**^ (0.89–0.95)	2 (0.36–1.13)	67 (0.93–0.98)	24 (0.75–0.91)	67 (0.89–0.96)	21 (0.81–0.96)
Persistent attempts to humiliate in front of colleagues	96	24^*^ (0.53–0.72)	42 (0.65–0.79)	61 (0.64–0.75)	7 (0.43–0.80)	67 (0.63–0.75)	2 (0.19–1.05)	50 (0.65–0.78)	18 (0.51–0.72)	49 (0.62–0.75)	16 (0.57–0.80)
Intimidating use of discipline or competence procedures	86	32^**^ (0.76–0.90)	50 (0.80–0.91)	76 (0.82–0.91)	8 (0.63–0.94)	82 (0.82–0.90)	2 (0.19–1.05)	62^**^ (0.84–0.93)	23 (0.69–0.87)	63 (0.83–0.92)	18 (0.65–0.87)
Undermining your personal integrity	52	18^**^ (0.36–0.56)	30 (0.45–0.60)	46 (0.46–0.58)	5 (0.25–0.64)	49 (0.45–0.57)	2 (0.36–1.13)	37 (0.46–0.60)	14 (0.36–0.59)	38 (0.46–0.60)	10 (0.29–0.54)
Destructive innuendo and sarcasm	77	28^**^ (0.65–0.82)	45 (0.70–0.83)	68 (0.71–0.82)	8 (0.55–0.89)	74^**^ (0.71–0.81)	2 (0.36–1.13)	54^**^ (0.71–0.83)	21 (0.63–0.83)	54 (0.69–0.81)	19 (0.69–0.89)
Verbal and non-verbal threats	68	30^**^ (0.64–0.81)	37 (0.55–0.70)	60 (0.62–0.74)	7 (0.47–0.83)	66^**^ (0.62–0.73)	2 (0.19–1.05)	49^**^ (0.62–0.75)	18 (0.52–0.74)	50 (0.63–0.76)	14 (0.47–0.71)
Making inappropriate jokes	59	24 (0.52–0.71)	33 (0.48–0.63)	50 (0.50–0.63)	7 (0.51–0.86)	57 (0.53–0.65)	1 (0.001–0.80)	42 (0.52–0.66)	16 (0.44–0.66)	44^**^ (0.54–0.67)	11 (0.32–0.57)
Persistent teasing	73	27^**^ (0.61–0.79)	42 (0.65–0.79)	62 (0.64–0.76)	9 (0.72–0.99)	70^**^ (0.66–0.77)	2 (0.36–1.13)	51 (0.67–0.79)	20 (0.59–0.79)	69^**^ (0.63–0.76)	17 (0.64–0.85)
Physical violence	11	5 (0.06–0.19)	6 (0.05–0.14)	9 (0.06–0.14)	1 (0.001–0.16)	10 (0.06–0.14)	1 (0.001–0.63)	6 (0.05–0.13)	4 (0.06–0.21)	7 (0.05–0.14)	3 (0.04–0.20)
Violence to property	39	13 (0.25–0.44)	23 (0.32–0.47)	34 (0.32–0.45)	4 (0.19–0.56)	38^**^ (0.33–0.45)	1 (0.001–0.63)	29 (0.33–0.47)	10 (0.23–0.44)	30 (0.34–0.48)	7 (0.16–0.39)
Withholding necessary information	79	27^**^ (0.62–0.80)	49 (0.77–0.89)	70^**^ (0.75–0.85)	7 (0.51–0.86)	77^**^ (0.75–0.84)	1 (0.05–0.94)	57 (0.75–0.86)	21 (0.64–0.84)	57^**^ (0.73–0.84)	17 (0.64–0.85)
Freezing out, ignoring, or excluding	85	29^**^ (0.67–0.84)	52 (0.84–0.94)	75 (0.81–0.90)	8 (0.59–0.92)	82^**^ (0.80–0.89)	2 (0.36–1.13)	61^**^ (0.82–0.92)	22 (0.67–0.86)	61 (0.79–0.89)	19 (0.73–0.92)
Unreasonable refusal of applications for leave, training, or promotion	81	27^**^ (0.62–0.80)	50 (0.80–0.91)	71 (0.76–0.86)	8 (0.63–0.94)	79^**^ (0.76–0.86)	2 (0.19–1.05)	59 (0.79–0.89)	21 (0.63–0.83)	58 (0.75–0.86)	19 (0.69–0.89)
Undue pressure to produce work	92	33^**^ (0.80–0.93)	55 (0.91–0.98)	82 (0.89–0.96)	9 (0.72–0.99)	89^**^ (0.89–0.95)	2 (0.36–1.13)	66^**^ (0.90–0.97)	25 (0.79–0.94)	67^**^ (0.89–0.96)	20 (0.77–0.94)
Setting of impossible deadlines	84	29^**^ (0.67–0.84)	51 (0.83–0.93)	74 (0.79–0.88)	9 (0.72–0.99)	81^**^ (0.79–0.88)	2 (0.36–1.13)	61 (0.82–0.92)	22 (0.66–0.85)	73 (0.81–0.91)	18 (0.65–0.87)
Constant undervaluing of efforts	85	29^**^ (0.68–0.85)	52 (0.83–0.93)	75 (0.81–0.90)	8 (0.59–0.92)	82^**^ (0.81–0.89)	2 (0.19–1.05)	61^*^ (0.82–0.91)	23 (0.70–0.88)	61 (0.79–0.89)	20 (0.75–0.93)
Persistent attempts to demoralize	85	29^**^ (0.68–0.85)	52 (0.84–0.94)	75 (0.81–0.90)	9 (0.68–0.97)	82 (0.81–0.89)	2 (0.36–1.13)	61^**^ (0.82–0.92)	23 (0.70–0.88)	62 (0.80–0.90)	19 (0.73–0.92)
Removal of areas of responsibility without consultation	77	26^**^ (0.85–0.76)	48 (0.75–0.87)	69 (0.73–0.84)	7 (0.43–0.80)	74 (0.71–0.81)	2 (0.36–1.13)	57^**^ (0.75–0.86)	19 (0.56–0.77)	54 (0.68–0.80)	19 (0.73–0.92)
Discrimination on racial or sexual grounds	73	21^**^ (0.46–0.65)	48 (0.76–0.88)	64 (0.67–0.78)	7 (0.55–0.89)	71 (0.67–0.78)	2 (0.19–1.05)	55^**^ (0.72–0.84)	17 (0.48–0.70)	51 (0.64–0.77)	18 (0.67–0.88)

** *P-value < 0.01*;

* *P-value < 0.05*.

### Personal Characteristics

Female Arabic Syrians who were <5'8 had a BMI of 25 and were 30 years old or younger were more likely to report specific bullying practices. Females were more likely than males to report the following forms of bullying: Attempts to minimize and devalue work (55 vs. 34%, *P* ≤ 0.01), continuous, unwarranted criticism and work monitoring (56 vs. 33%, *P* ≤ 0.01), continuous attempts to humiliate in front of colleagues (42 vs. 24 %, *P* ≤ 0.05), Use of threatening discipline or competency processes (50 vs. 32%, *P* ≤ 0.01), Undermining personal integrity (30 vs. 18%, *P* ≤ 0.01), Sarcasm and destructive innuendo (45 vs. 28%, *P* ≤ 0.01), Threats, both verbal and nonverbal (37 vs. 30%, *P* ≤ 0.01), continual teasing (42 vs. 27%, *P* ≤ 0.01), omitting crucial data (49 vs. 27%, *P* ≤ 0.01), Freezing out, ignoring, or excluding (52 vs. 29%, *P* ≤ 0.01), Unreasonable denials of leave, training, and promotion applications (50 vs. 27%, *P* ≤ 0.01), Unreasonable pressure to perform work (55 vs. 33%, *P* ≤ 0.01), Establishment impossible deadlines (51 vs. 29%, *P* ≤ 0.01), Constant efforts undervaluing (52 vs. 29%, *P* ≤ 0.01), continuous demoralizing attempts (52 vs. 29%, *P* ≤ 0.01), Removal of areas of responsibility without consultation (48 vs. 26%, *P* ≤ 0.01), Discrimination based on race or sexual orientation (48 vs. 21%, *P* ≤ 0.01). The Arabic Syrian group has reported more bullying behaviors than the Non-Arabic-Syrian group: Omitting crucial data (70 vs. 7%, *P* ≤ 0.01). The ≤30 age group reported the following bullying behaviors more than the >30 age group: attempts to minimize and devalue work (89 vs. 3%, *P* ≤ 0.01), continuous, unwarranted criticism and work monitoring (90 vs. 2%, *P* ≤ 0.01), Sarcasm and Destructive innuendo (74 vs. 2%, *P* ≤ 0.01), Threats, both verbal and non-verbal (66 vs. 2%, *P* ≤ 0.01), continual teasing (70 vs. 2%, *P* ≤ 0.01), Property-related violence (38 vs. 1%, *P* ≤ 0.01), Omitting crucial data (77 vs. 1%, *P* ≤ 0.01), Freezing out, ignoring, or excluding (82 vs. 2%, *P* ≤ 0.01), Unreasonable denials of leave, training and promotion application (79 vs. 2%, *P* ≤ 0.01), Unreasonable pressure to perform work (89 vs. 2%, *P* ≤ 0.01), Establishment of impossible deadlines (81 vs. 2%, *P* ≤ 0.01), Constant efforts undervaluing (82 vs. 2%, *P* ≤ 0.01).

Between group differences for the < 5'8 group in comparison to the ≥5'8 group were: Use of threatening discipline or competency processes (62 vs. 23%, *P* ≤ 0.01), Sarcasm and destructive innuendo (54 vs. 21%, *P* ≤ 0.01), Threats, both verbal and non-verbal (49 vs. 18%, *P* ≤ 0.01),

Freezing out, ignoring, or excluding (61 vs. 22%, *P* ≤ 0.01), Unreasonable pressure to perform work (66 vs. 25%, *P* ≤ 0.01), Continuous efforts undervaluing (61 vs. 23%, *P* ≤ 0.05), Continuous demoralizing attempts (61 vs. 23%, *P* ≤ 0.01), Removal of areas of responsibility without consultation (57 vs. 19%, *P* ≤ 0.01), Discrimination based on race or sexual orientation (55 vs. 17%, *P* ≤ 0.01). Between group differences for the BMI ≤ 25 group in comparison to the <25 group were: Making inappropriate jokes (44 vs. 11%, *P* ≤ 0.01), continual teasing (69 vs. 17%, *P* ≤ 0.01), Omitting crucial data (57 vs. 17%, *P* ≤ 0.01), Unreasonable pressure to perform work (67 vs. 20%, *P* ≤ 0.01).

### Professional Characteristics

General medical graduates and PGY 2-PGY-6 responders reported more specific bullying behaviors than private medical graduates and PGY 1 participants. General medical sector graduates, in comparison to private medical sector graduates, reported more continual teasing (65 vs. 5%, *P* ≤ 0.01), Omitting crucial data (71 vs. 6%, *P* ≤ 0.01), Unreasonable pressure to perform work (83 vs. 6%, *P* ≤ 0.01), Establishment of impossible deadlines (75 vs. 7%, *P* ≤ 0.01), PGY-2-PGY-6, compared to PGY1, reported more attempts to minimize and devalue work (93 vs. 86%, *P* ≤ 0.01), Continuous and unwarranted criticism and work monitoring (44 vs. 26%, *P* ≤ 0.01), Persistent attempts to humiliate in front of colleagues (32 vs. 17%, *P* ≤ 0.01), Use of threatening discipline or competency processes (39 vs. 25%, *P* ≤ 0.01), Undermining personal integrity (25 vs. 12%, *P* ≤ 0.01), Sarcasm and destructive innuendo (36 vs. 21%, *P* ≤ 0.01), Threats, both verbal and non-verbal threats (30 vs. 19%, *P* ≤ 0.01), Jokes making (27 vs. 14%, *P* ≤ 0.01), Continual teasing (36 vs. 18%, *P* ≤ 0.01), Omitting crucial data (36 vs. 22%, *P* ≤ 0.01), Freezing out, ignoring, or excluding (37 vs. 26%, *P* ≤ 0.01), Unreasonable denials of leave, training, or promotion applications (36 vs. 24%, *P* ≤ 0.01), Unreasonable pressure to perform work (48 vs. 26%, *P* ≤ 0.01), Continuous efforts undervaluing (39 vs. 24%, *P* ≤ 0.01), Persistent attempts to demoralize (40 vs. 24%, *P* ≤ 0.01), Removal of areas of responsibility without consultation (36 vs. 20%, *P* ≤ 0.01), Discrimination based on race or sexual orientation (34 vs. 18%, *P* ≤ 0.01). On contrast PGY 1 in comparison to PGY-2-PGY-6 reported more Property-related violence (12 vs. 11%, *P* ≤ 0.01), Establishment of impossible deadlines (76 vs. 6%, *P* ≤ 0.01) (Table 4).

**Table 4:** Percentages of bullying behaviors by professional characteristics.

	**Medical student**		**PGY**	
	**General sector (*n* = 105) 95%CI (lower-upper)**.	**Private sector (*n* = 161). 95%CI (lower-upper)**	**PGY-1 (*n* = 84) 95%CI (lower-upper)**	**PGY-2- PGY-6 (*n* = 130) 95%CI (lower-upper)**
Subjected to bullying	79 (0.85–0.93)	60 (0.58–0.96)	79^**^ (0.70–0.88)	88 (0.82–0.93)
attempts to belittle and undermine work	93 (0.89–0.96)	86 (0.70–1.01)	86^**^ (0.78–0.93)	93 (0.88–0.97)
Persistent and unjustified criticism and monitoring of work	94 (0.90–0.96)	77 (0.58–0.96)	26^**^ (0.76–0.92)	44 (0.89–0.98)
Persistent attempts to humiliate in front of colleagues	62 (0.63–0.75)	5 (0.41–0.85)	17^**^ (0.46–0.67)	32 (0.61–0.77)
Intimidating use of discipline or competence procedures	78 (0.82–0.91)	6 (0.52–0.92)	25^**^ (0.73–0.90)	39 (0.75–0.86)
Undermining your personal integrity	47 (0.46–0.59)	3 (0.14–0.58)	12^**^ (0.27–0.48)	25 (0.43–0.61)
Destructive innuendo and sarcasm	69 (0.72–0.82)	5 (0.41–0.85)	21^**^ (0.60–0.80)	36 (0.68–0.83)
Verbal and non-verbal threats	61 (0.62–0.74)	5 (0.36–0.81)	19^**^ (0.50–0.71)	30 (0.55–0.72)
Making inappropriate jokes	45 (0.54–0.66)	3 (0.18–0.63)	14^**^ (0.35–0.57)	27 (0.48–0.65)
Persistent teasing	65^**^ (0.67–0.78)	5 (0.36–0.81)	18^**^ (0.48–0.70)	36 (0.68–0.83)
Physical violence	10 (0.07–0.15)	0 (0.001–0.14)	3 (0.03–0.15)	5 (0.04–0.15)
Violence to property	36 (0.34–0.46)	2 (0.03–0.41)	12^**^ (0.28–0.49)	11 (0.15–0.30)
Withholding necessary information	71^**^ (0.74–0.84)	6 (0.52–0.92)	22^**^ (0.61–0.81)	36 (0.69–0.84)
Freezing out, ignoring, or excluding	76 (0.80–0.89)	7 (0.64–0.99)	26^**^ (0.76–0.92)	37 (0.71–0.85)
Unreasonable refusal of applications for leave, training, or promotion	73 (0.77–0.86)	5 (0.47–0.89)	24^**^ (0.68–0.86)	36 (0.68–0.83)
Undue pressure to produce work	83^**^ (0.89–0.96)	6 (0.58–0.96)	26^**^ (0.84–0.96)	48 (0.83–0.94)
Setting of impossible deadlines	75^**^ (0.79–0.88)	7 (0.70–1.01)	76^**^ (0.70–0.88)	6 (0.73–0.86)
Constant undervaluing of efforts	24 (0.81–0.90)	38 (0.52–0.92)	24^**^ (0.68–0.86)	39 (0.76–0.89)
Persistent attempts to demoralize	76 (0.81–0.90)	6 (0.58–0.96)	24^**^ (0.69–0.87)	40 (0.77–0.90)
Removal of areas of responsibility without consultation	70 (0.72–0.83)	38 (0.36–0.81)	20^**^ (0.55–0.75)	36 (0.67–0.82)
Discrimination on racial or sexual grounds	67 (0.69–0.80)	5 (0.31–0.77)	18^**^ (0.48–0.70)	34 (0.63–0.79)

** *P-value < 0.01*;

^*^P-value < 0.05.

## Discussion

### Summary of the Findings

This study explores the frequency of bullying in the Syrian Graduate Medical Education System across various demographic and category groups. Our findings support the previously reported findings in the literature: many young physicians are bullied, with females being more likely to be bullied (6, 7). This might be explained by the fact that most females are more emotional and sensitive than males and are more likely to experience a subjective sense of victimization. In addition, recent systematic evaluations of prior research revealed a wide range of bullying prevalence (30–95%) among young doctors in the workplace (8); this might be explained by the numerous bullying evaluation instruments utilized in this research to estimate the prevalence of bullying in its many manifestations among coworkers in general and medical staff in particular. Furthermore, researchers tend to employ the Leymann criterion, Mikkelsen and Einarsen's criterion, or the cut-off score to pick the approaches (9–11).

There was no big statistical difference between the general and private medical sectors regarding bullying experience at work. However, the general medical sector graduates tended to report specific bullying behaviors in the questionnaire than the private medical sector graduates, which could be attributed to the unbalanced sample size between the two groups. The same interpretation could be made for the result that Syrian doctors were subjected to bullying generally and racist discrimination, specifically ten times more than non-Syrian doctors, which leads to a misleading conclusion.

The current study's findings are congruent with those of comparable national surveys conducted in other parts of the world, demonstrating that a significant number of young physicians are bullied at work. This is demonstrated by the findings of a meta-analysis that looked at a large number of studies (*n* = 44,878, *k* = 15 samples) and found that using the combination technique resulted in a weighted prevalence of 3.7% bullying (7), implying that young physicians were bullied more frequently than older doctors and consultants. This might be due to characteristics peculiar to junior physicians, such as a rigorous medical hierarchy that necessitates fast-paced and unpredictable labor and the frequent practice of 'education by humiliation' throughout medical school (3, 12) because junior doctors in different countries are subjected to overwhelming resident duty and training hours, and amount of clinical supervision (13). In addition, in the medical field, there is a conventional power structure in which junior physicians are at the bottom of the pecking order, leading to being more vulnerable to bullying. On the other side, perceptions and interpretations have been proposed to vary with age and maturity, which might explain why older doctors are less prone to notice bullying (14).

The findings of this study on unpleasant experiences in different specialties were flawed by insufficient sampling for each group, which may necessitate further inquiry by targeting each group individually in a future study to ensure obtaining a representative sample for all medical specialties.

The observation that female doctors reported bullying exposure at work more often than males is expected, as other studies published in the literature came with the same result (5, 6, 15–17). Some speculated that this was because men and women interpret workplace bullying differently, with men seeing it as a managerial style and women seeing it as a threat (18). Others say that because women are allowed smaller ranges of acceptable conduct, departures from established norms may result in negative feedback and increase their risk of workplace bullying (19, 20). Our results recommend supporting young doctors by preventing bullying, this could be achieved by launching educational programs and increasing cultural awareness among medical sector about the bad consequences on bullying on young doctors and the benefit of bullying prevention in improving the quality of medical training for doctors.

### Limitations of the Study

The present study has several limitations. First, despite being a cost-effective and practical technique, the cross-sectional research design cannot demonstrate causality. Furthermore, because the study was done among presently employed young physicians, individuals who had been badly impacted by bullying to resigning or developing sickness needing long-term leave may have been omitted from the study. As a result, workplace bullying's incidence may have been underestimated.

Apart from that, the generalizability of this study was increased by using universal sampling and reaching a response rate of 99%, which is greater than the typical response rate for organizational research surveys (21). Reporting a bias in studies is important, however, in this study, we excluded sampling bias, response bias, non-response bias, acquiescence bias, and order bias which is a potential limitation of this study.

Despite the previously mentioned limitations, several steps were taken to increase the robustness of this study. For example; they are sampling from multiple study sites and applying universal sampling procedures to elevate the external validity of study results, conducting a priori sample size calculation to make sure that the study is powerful, and using the validated instrument in addition to adjusting for potential confounders in the final model to increase the internal validity of study results.

## Conclusion

Our research demonstrates that bullying is prevalent in the Syrian Graduate Medical Education System. Therefore, many recommended measures should be used to increase doctor-to-doctor communication to avoid lowering the quality of patient care, especially because practically all Syrian doctors are emigrating to neighboring countries due to their low economic status.

## Data Availability Statement

The raw data supporting the conclusions of this article will be made available by the authors, without undue reservation.

## Ethics Statement

The studies involving human participants were reviewed and approved by the Ethics Committee of Aleppo University; Ibn Al-Nafees Hospital. The patients/participants provided their written informed consent to participate in this study.

## Author Contributions

SaS: conceptualization, methodology, formal analysis, and writing—original draft, and review and editing. ShS: conceptualization and writing—original draft. MA and HB: writing—original draft and review and editing. AH, KM, NH, EA, LS, LK, AB, AM, DM, FA, and SA: writing—review and editing. MH: reviewing the final draft of the manuscript. NE: conceptualization and writing—original draft. All authors contributed to the article and approved the submitted version.

## Conflict of Interest

The authors declare that the research was conducted in the absence of any commercial or financial relationships that could be construed as a potential conflict of interest.

## Publisher's Note

All claims expressed in this article are solely those of the authors and do not necessarily represent those of their affiliated organizations, or those of the publisher, the editors and the reviewers. Any product that may be evaluated in this article, or claim that may be made by its manufacturer, is not guaranteed or endorsed by the publisher.

## References

[1] Silver HK. Medical students and medical school. JAMA. (1982) 247:309–10. 10.1001/jama.247.3.309 7054531

[2] Swearer S Collins A Berry B. Bullying. Encyclopedia of Human Behavior. 2nd ed. Elsevier Inc. (2012). p. 417–22. 10.1016/B978-0-12-375000-6.00077-X

[3] Leisy HB Ahmad MJ. Altering workplace attitudes for resident education (AWARE): discovering solutions for medical resident bullying through literature review. BMC. (2016) 16:127. 10.1186/s12909-016-0639-8 27117063 PMC4847214

[4] Daugherty SR Baldwin DC Jr Rowley BD. Learning, satisfaction, and mistreatment during medical internship: a national survey of working conditions. JAMA. (1998) 279:1194–9. 10.1001/jama.279.15.1194 9555759

[5] Chadaga AR Villines D Krikorian AJ. Bullying in the American graduate medical education system: a national cross-sectional survey. PLoS ONE. (2016) 11:e0150246. 10.1371/journal.pone.0150246 26982705 PMC4794154

[6] Ling M Young CJ Shepherd HL Mak C Saw RPJ. Workplace bullying in surgery. World J Surg. (2016) 40:2560–6. 10.1007/s00268-016-3642-7 27624759

[7] Zapf D Escartin J Scheppa-Lahyani M Einarsen SV Hoel H Vartia M. Harassment in the workplaceitheory R, practice. In: Empirical Findings on Prevalence and Risk Groups of Bullying in the Workplace, Vol. 3. Taylor & Francis (2020). p. 105–62. 10.1201/9780429462528-5

[8] Samsudin EZ Isahak M Rampal SJ. The prevalence, risk factors and outcomes of workplace bullying among junior doctors: a systematic review. Eur J Work Organ Psychol. (2018) 27:700–18. 10.1080/1359432X.2018.1502171

[9] Leymann H. Mobbing and psychological terror at workplaces. Violence Vict. (1990) 5:119–26. 10.1891/0886-6708.5.2.119 2278952

[10] Leymann H. The content and development of mobbing at work. Eur J Work Organ Psychol. (1996) 5:165–84. 10.1080/1359432960841485312350256

[11] Mikkelsen EG Einarsen S. Bullying in Danish work-life: prevalence and health correlates. Eur J Work Organ Psychol. (2001) 10:393–413. 10.1080/13594320143000816

[12] Paice E Smith DJ. Bullying of trainee doctors is a patient safety issue. Clin Teach. (2009) 6:13–17. 10.1111/j.1743-498X.2008.00251.x

[13] Temple JJ. Resident duty hours around the globe: where are we now? BMC. (2014) 14:S8. 10.1186/1472-6920-14-S1-S8 25559277 PMC4304287

[14] Crutcher RA Szafran O Woloschuk W Chatur F Hansen CJ. Family medicine graduates' perceptions of intimidation, harassment, and discrimination during residency training. BMC. (2011) 11:88. 10.1186/1472-6920-11-88 22018090 PMC3258190

[15] Aykut G Efe EM Bayraktar S Sentürk S Başegmez I Özkumit Ö. Mobbing exposure of anaesthesiology residents in Turkey. Turk J Anaesthesiol Reanim. (2016) 44:177–89. 10.5152/TJAR.2016.79446 27909591 PMC5019868

[16] Fnais N al-Nasser M Zamakhshary M Abuznadah W Al-Dhukair S Saadeh M. Prevalence of harassment and discrimination among residents in three training hospitals in Saudi Arabia. Ann Saudi Med. (2013) 33:134–9. 10.5144/0256-4947.2013.134 23563000 PMC6078628

[17] Hills DJ Joyce CM Humphreys JS. A national study of workplace aggression in Australian clinical medical practice. Med J Aust. (2012) 197:336–40. 10.5694/mja12.10444 22994831

[18] Simpson R Cohen C. Dangerous work: the gendered nature of bullying in the context of higher education. Gender Work Organiz. (2004) 11:163–86. 10.1111/j.1468-0432.2004.00227.x

[19] Babcock L Laschever S. Women Don't Ask: Negotiation and the Gender Divide. Princeton University Press (2003). 10.1515/9780691212845

[20] Gilbert JA Raffo DM Sutarso TJ. Gender, conflict, and workplace bullying: is civility policy the silver bullet? J Manag Issues. (2013) 25:79–98. Available online at: https://www.researchgate.net/publication/320146168_Gender_conflict_workplace_bullying_Is_civility_policy_the_silver_bullet

[21] Baruch Y Holtom BC. Survey response rate levels and trends in organizational research. Hum Relat. (2008) 61:1139–60. 10.1177/0018726708094863

